# Contribution of both positive selection and relaxation of selective constraints to degeneration of flyability during geese domestication

**DOI:** 10.1371/journal.pone.0185328

**Published:** 2017-09-25

**Authors:** Ye Wang, Yaodong Hu, Daqian He, Shiyi Chen, Siming Li, Dan Lan, Peng Ren, Zhenping Lin, Yiping Liu

**Affiliations:** 1 Farm Animal Genetic Resources Exploration and Innovation Key Laboratory of Sichuan Province, Sichuan Agricultural University, Chengdu Campus, Chengdu, Sichuan, PR China; 2 Institute of Animal Husbandry & Veterinary Sciences, Shanghai Academy of Agricultural Sciences, Shanghai, PR China; 3 Institute of Animal Husbandry and Veterinary Sciences, Jiangxi Academy of Agricultural Science, Nanchang, PR China; 4 Shantou Baisha Research Institute of Original Species of Poultry and Stock, Shantou, Guangdong, PR China; National Cheng Kung University, TAIWAN

## Abstract

Flyability is the most discrepant trait between modern-day geese and their wild ancestors, and the degeneration of flyability is a key marker of the successful domestication of wild geese. In light of the relatively short history of domestic geese, intense artificial selection is thought to play an important role in the degeneration of flyability. However, the underlying mechanism behind this phenomenon has seldom been investigated. In this study, we applied a molecular evolutionary approach to the evaluation of partial breeds of domestic geese in order to look for genes involved in the selection pressure toward degeneration of flyability. The haplotype networks, pairwise fixation index (*F*_*ST*_) values, and analysis of molecular variance results all clearly illustrated a population variance between Landes geese and partial Chinese domestic geese. We also detected signatures of positive artificial selection in the *COX2* and *COX3* genes, and related selection in the *HBB* gene. Our results support the independent origins of partial European domestic geese and Chinese domestic geese. In addition, both positive artificial selection and the relaxation of functional constraints appeared to play important roles in the degeneration of flyability in domestic geese.

## Introduction

Flyability is the most discrepant trait between modern-day geese and their wild ancestors. Wild geese were one of the most celebrated high-altitude performers for their stamina and the heights they achieved. Bar-headed geese (*Anser indicus*) were known to pass over the Himalayas in only one day and to climb between 4,000 and 6,000 m in 7–8 h [1]. In contrast, modern-day domestic geese perform poorly at flying, and most of them have lost the ability to gain altitude. The degeneration of flyability is a key marker of the successful domestication of geese, since their ability to maintain flight would be detrimental to their artificial breeding. Recent archaeological discoveries have indicated that geese domestication began at around 1,700 bce, which means that domestic geese have been in existence for less than 4,000 years [2–4]. It is remarkable that these birds had lost their ability to fly in such a relatively short time span without significant accompanying morphological changes. So far, few studies have concentrated on degeneration of flyability, and little is known about the physiological and molecular basis of the phenomenon. Historically, artificial selection has been considered to be the main driving force behind trait changes occurring during domestication; for instance, the coat color of swine and the semi-dwarf phenotype of rice [5]. Therefore, we speculated that artificial selection might be responsible for the loss of flyability in geese during their domestication.

Flight is one of the most energy-consuming movements in animals [6]. Studies performed on barnacle geese (*Branta leucopsis*) and bar-headed geese (*A*. *indicus*) revealed that the flying state required 10–20-fold more oxygen (O_2_) than the walking state [7, 8]. As a specialized flying mammal, the metabolic rate of bats is 3–5 times higher than that of similarly sized terrestrial mammals during strenuous exercise [9, 10]. Typically, the mitochondrial respiratory chain produces almost 95% of the adenosine triphosphate required for movement [11]. Cytochrome *c* oxidase (COX, also named Complex IV) is the terminal oxidase of the mitochondrial respiratory chain and yields twice the amount of energy of the other two steps in electron transport. Furthermore, COX has been found to represent the rate-limiting step in intact cells during respiration. This reinforces the assumption that COX plays a large role in control of the respiratory chain and thus of global energy production [12–14]. *COX* is composed of nuclear genes and mitochondrial genes, and three mitochondrial genes are encoded within its catalytic core (viz., *COX1*, *COX2*, and *COX3*) [15]. A mitochondrial study performed on avian subjects demonstrated that functional constraints caused by limited locomotion play an important role in the evolution of mitochondrial DNA (mtDNA) [16]. The authors of that study found that with the degeneration of flyability, the mtDNA accumulated more nonsynonymous nucleotide substitutions, where the relaxation of selective constraints was deemed to be the dominant driving force [16]. In view of this, the *COX* genes could reveal some signatures of selection following the obvious degeneration of flight in domestic geese. The efficiency of O_2_ transport is another critical factor affecting the locomotion of birds. Hemoglobin (Hb), the carrier of O_2_ in blood, is a tetrameric molecule composed of two α (or α-like) chains and two β (or β-like) chains that combine to produce major isoHb (α^A^β^A^) and minor isoHb (α^D^β^A^) molecules. The α^D^, α^A^, and β^A^ chains are encoded by the *HBA1*, *HBA2*, and *HBB* genes, respectively [17]. Studies performed on waterfowls, frogs, and deer mice suggest that Hb can adapt to the high-altitude conditions found in nature [18–20]. In a comparative study [20], differences were found between the Hb molecules of highland and lowland waterfowls. Obviously, the Hb genes are sensitive to O_2_ pressure and the flyability of birds. Hence, we surmised that the Hb genes may reveal trace evidence of the selection that affects the flyability of domestic geese.

Poor flying performance in domestic geese is correlated with less energy consumption and more energy storage, and is an important prerequisite for the successful domestication of geese. Although it is a reasonable assumption that artificial selection had affected the flyability of domestic geese, the target and type of selection involved remains unclear. Therefore, to provide direct evidence of selection pressure on the loss of flyability during domestication and to identify the type of selection utilized, we focused on the comparison of two mitochondrial genes (*COX2* and *COX3*) and three nuclear genes (*HBA1*, *HBA2*, and *HBB*) in domestic geese and their wild ancestors. Chinese and European domestic geese reportedly had originated independently from the swan goose (*Anser cygnoides*) and the Greylag Landaise goose (*Anser anser*), respectively [21]. It is interesting that both lineages had lost their flyability during domestication, which reveals a parallel evolutionary tendency. Thus, another goal of this study was to look for any differences in the above-mentioned genes between these two lineages at both the population and genetic levels.

## Materials and methods

### Ethics statement

The protocols used in this study were approved by the Institutional Animal Care and Use Committee of Sichuan Agricultural University (Permit No. DKY-S20131105).

### Sample collection

Blood samples were collected from 172 individuals representing the following: one breed of European domestic geese (viz., the Landaise goose, divided into the gray plumage Landes group (G) and the spotted feather Landes group (H)); four breeds of Chinese domestic geese (viz., the Lion-head goose (S), Sichuan white goose (SC), Eastern Zhejiang white goose (Z), and Zi goose (Zi)); and two wild ancestors (viz., *A*. *cygnoides* (B) and *A*. *anser* (Hui)). To avoid accidental collection from related individuals, we obtained samples from geese with clear pedigrees only. The information obtained from the samples is presented in S1 Table.

### Primer design, PCR amplification, and sequencing

Genomic DNA was isolated from the blood samples using a standard phenol-chloroform extraction protocol. Primers for *COX2* and *COX3* were designed using the known mitochondrial genome of *A*. *anser* (GenBank Accession No. NC_011196.1). The primers used to amplify the partial sequence of *EVC2P* were designed using the class II-related endogenous retrovirus of *Anser albifrons* (GenBank Accession No. AY820076.1) [22]. Amplification of *HBA1*, *HBA2*, and *HBB* was performed according to the method described by McCracken et al. [23]. All primers used in this study are listed in S2 Table.

Polymerase chain reaction (PCR) amplification was performed using a 50 μL reaction mixture containing 100 ng of DNA, 10 mmol/L of Tris-HCl (pH 8.3), 2.5 mmol/L of MgCl_2_, 50 mmol/L of KCl, 10 mM of each dNTP, 10 pmol/L of each primer, and 1 unit of Taq polymerase (Takara, Dalian, China). The PCR protocol included a 7 min preheat at 95°C; followed by 35 cycles of 30 s at 94°C, 30 s at 56–68°C, 1–2 min at 72°C; and a final extension of 10 min at 72°C. The PCR products were sequenced at Sangon Biotech (Shanghai, China) using an ABI 3730 sequencer (Applied Biosystems, Foster City, CA, USA) and the primers listed in S2 Table. Samples with rare variants were verified by resequencing.

### Data analyses

All sequences acquired in this study were edited and assembled using BioEdit version 7.0 [24]. Multiple sequence alignment was performed using ClustalX 2.0 [25], and variable sites at each gene were analyzed using MEGA6 [26]. The number of haplotypes (H), haplotype diversity (Hd), nucleotide diversity (π), average number of nucleotide differences (K), Tajima’s *D* test, Fu and Li’s *D* test, and Fu’s *Fs H* test were determined for each population, using DnaSP 5.0 [27]. The haplotype networks were constructed using the pegas, plyr, and reshape packages of R program, and were modified manually for improved illustration.

Adaptation to various selection pressures always increases the degree of variance among subpopulations. Thus, we applied the *F*_*ST*_ value to measure the degree of variance for each population, and detected outlier loci based on the *F*_*ST*_ value at the single nucleotide polymorphism (SNP) level. The computation was performed from 10,000 simulations with 100 demes per group, under the assumption of a hierarchical island model, by ARLEQUIN version 3.5 [28]. To determine the amount of genetic variance at each hierarchical level, we also performed an analysis of molecular variance (AMOVA) based on the haplotype frequencies and pairwise differences in ARLEQUIN. To further study the patterns of polymorphism and divergence, Hudson-Kreitman-Aguade (HKA) tests, allowing comparison with the neutral model, were implemented for each gene by using DnaSP 5.0. The ratio of nonsynonymous (*d*_*N*_) to synonymous (*d*_*S*_) substitutions and standard errors were calculated using 1,000 bootstrap replicates via MEGA6, and statistical significance was evaluated using the *Z* test.

## Results

### Sequence variation and molecular diversity

Whole DNA sequences of *COX2*, *COX3*, *HBA1*, and *HBA2* as well as partial sequences of *HBB* (intron 1, exon 2, and intron 2) and *EVC2P* were analyzed in this study. We found that there were relatively fewer polymorphic sites in the *COX2* and *COX3* genes of the Zi, *A*. *cygnoides*, and G populations than in those of the other populations, as well as fewer variable sites in *COX3* than in *COX2*. A relatively large number of mutations were found in *EVC2P*, with a low divergence among all populations except *A*. *anser* (S3 Table). In addition, we screened several population-specific mutations using the amino acid sequences. Mutation E88K of *COX2* occurred with a high frequency in Landaise geese and moderate frequency in *A*. *cygnoides* and *A*. *anser*, but was hardly found in Chinese domestic geese (S1 File). No other population-specific amino acid mutation was found among the other genes (S2–S5 Files).

**Fig 1:**
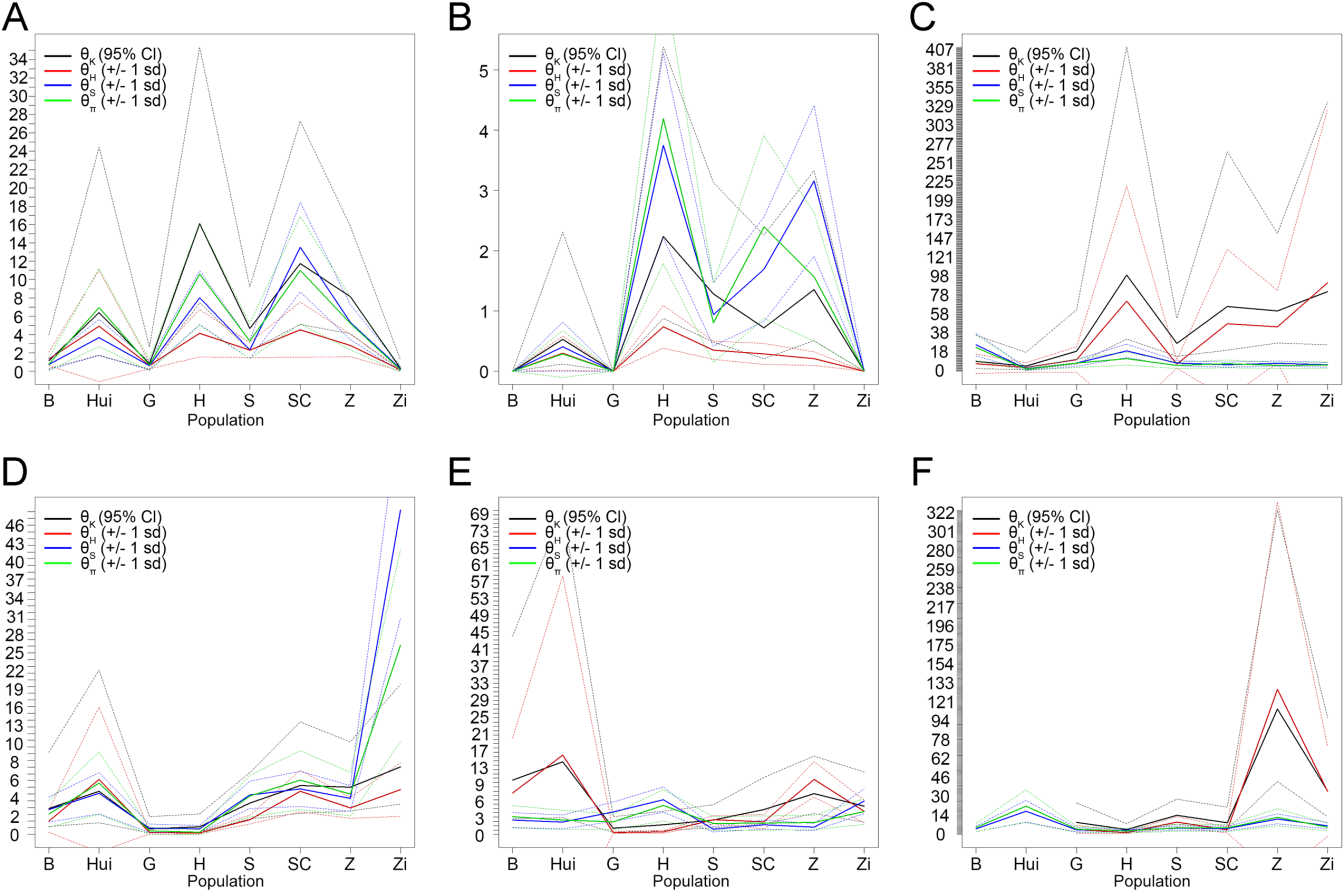
Molecular diversity indices of six gene fragments. (A) *COX2*, (B) *COX3*, (C) *EVC2P*, (D) *HBA1*, (E) *HBA2*, and (F) *HBB*. θ_*k*_, average number of nucleotide differences; θ_*H*_, haplotype diversity; θ_*S*_, number of polymorphic sites; θ_π_, nucleotide diversity.

The molecular diversity indices (θ_π_ and θ_*s*_) in *COX2* of B, G, S, and Zi were obviously lower than those in the other populations (Fig 1a). A similar trend of a smaller average θ value was observed for *COX3* (Fig 1b). For the *HBA1*, *HBA2*, *HBB*, and *EVC2P* fragments, an even distribution of θ_π_ and θ_*s*_ was observed among the populations, where the θ values for *HBB* and *EVC2P* were much higher than those for *HBA1* and *HBA2* (Fig 1c–1f). The nucleotide diversity of *EVC2P* was much richer and more regular than that of the other genes, with its mean π value being about 10-fold larger. Extremely low π values were identified for *COX2*, *COX3*, and *HBA1*, whereas the π values of *HBA2* and *HBB* were relatively higher and more stable.

### Population differentiation due to genetic structures

**Fig 2:**
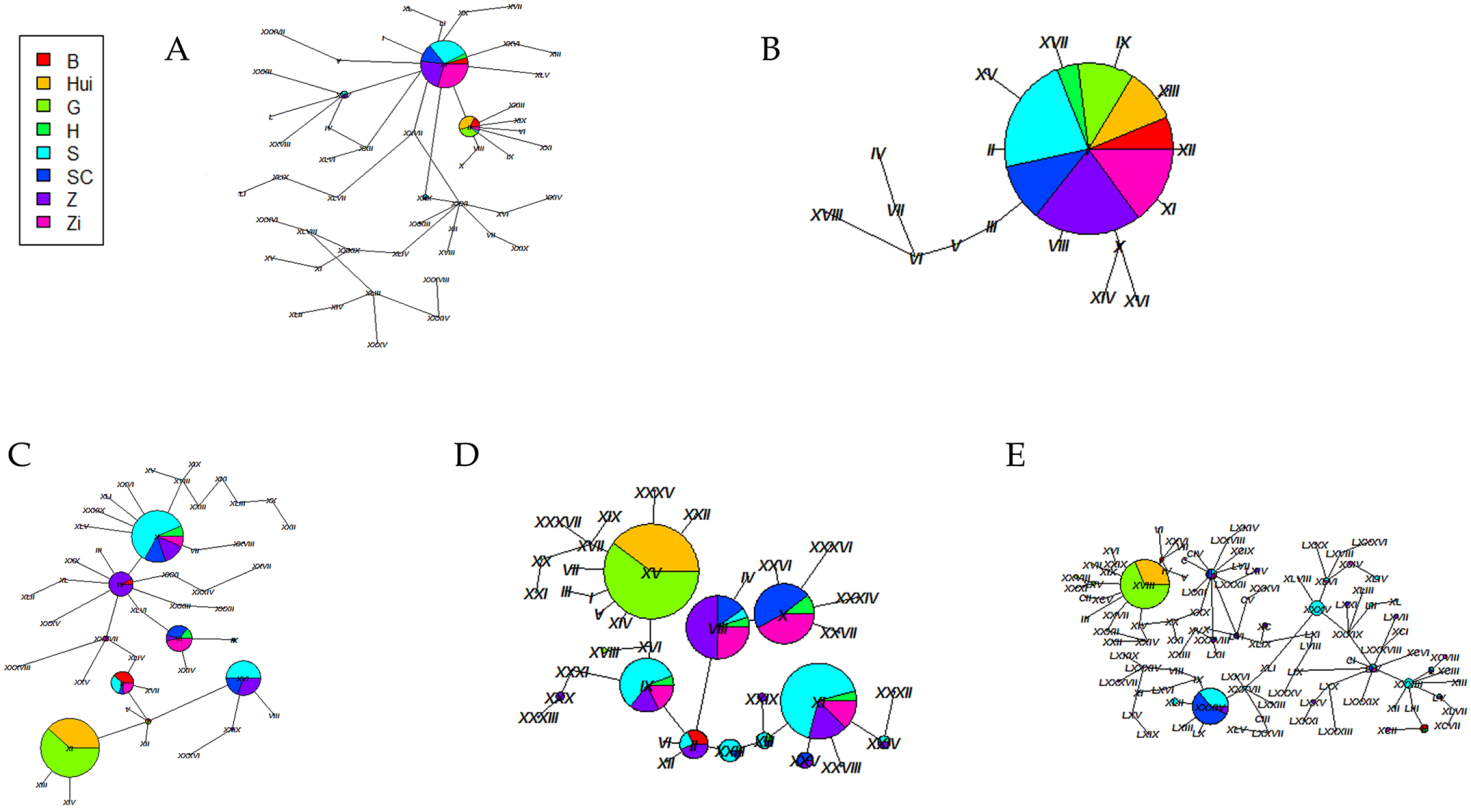
Haplotype networks of five genes across each population. (A) *COX2*, (B) *COX3*, (C) *HBA1*, (D) *HBA2*, and (E) *HBB*.

A total of 49, 16, 44, 44, and 102 haplotypes were detected in the *COX2*, *COX3*, *HBA1*, *HBA2*, and *HBB* genes, respectively. The networks of the two mitochondrial genes (*COX2* and *COX3*) showed *COX3* as being more conservative than *COX2*. A distinct variance in main haplotypes between the wild ancestors and domestic geese was observed for *COX2* (Fig 2a and 2b). Divergent clusters of main haplotypes in *HBA1*, *HBA2*, and *HBB* were found simultaneously in the European geese (Hui and G) and Chinese geese (Fig 2c–2e).

**Fig 3:**
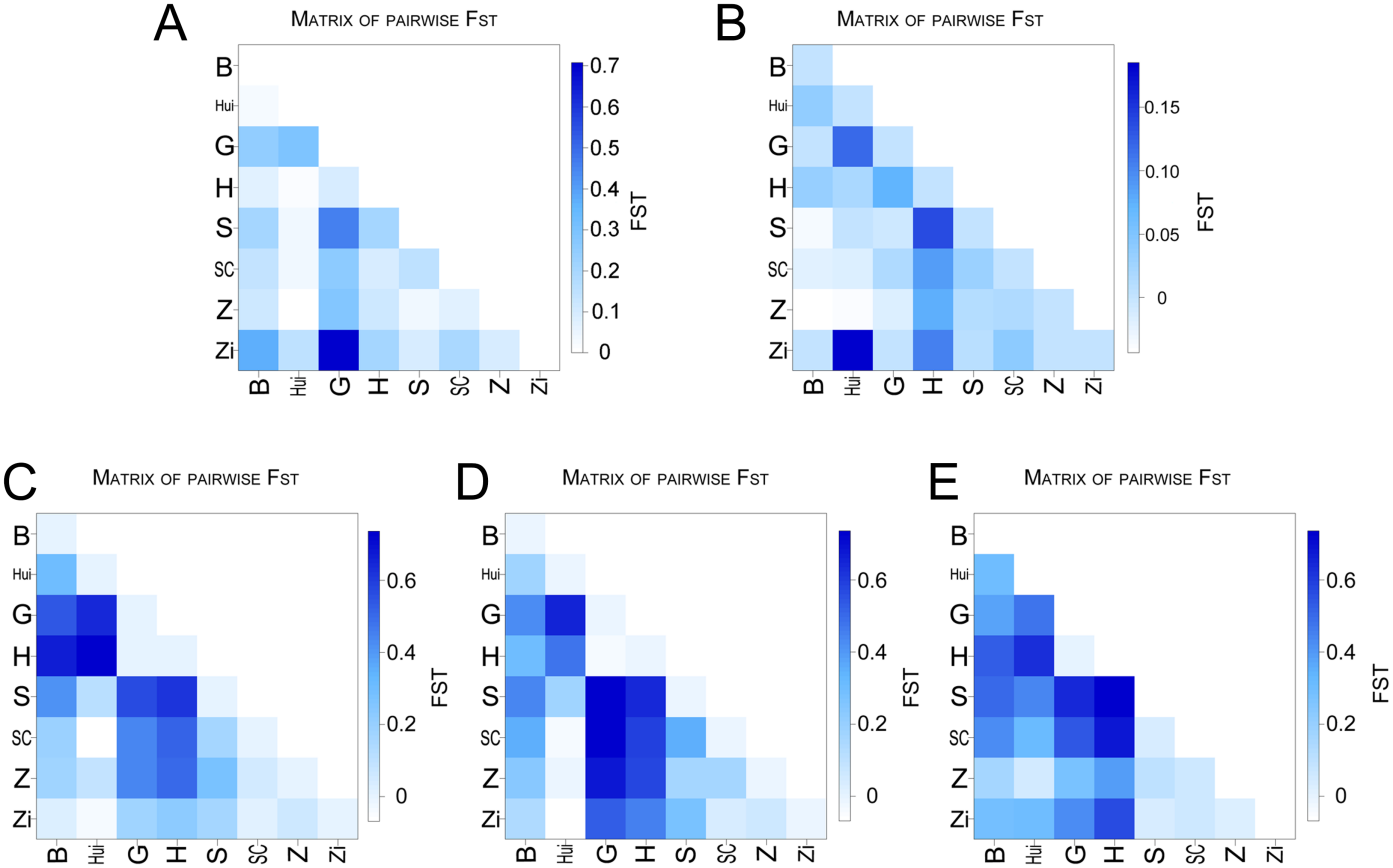
Matrix of pairwise *F*_*ST*_ for each gene across populations. (A) *COX2*, (B) *COX3*, (C) *HBA1*, (D) *HBA2*, and (E) *HBB*.

To further estimate the degree of population variance, we calculated the pairwise *F*_*ST*_ value. Interestingly, compared with *COX2* and *COX3*, significant differences in the three karyogenes *HBA1*, *HBA2*, and *HBB* were found between the Chinese geese populations and the Landaise geese (G and H) (Fig 3c–3e). Furthermore, the *F*_*ST*_ values of *COX3* (0–0.15) for each population were much lower than those of *COX2* (0–0.7), which again proved that *COX3* was more evolutionarily conserved than *COX2* (Fig 3a and 3b).

The hierarchical AMOVA was conducted, using the percentages of total genetic variation partitioned among groups (Chinese geese and *A*. *cygnoides*, Landaise geese and *A*. *anser*), among populations within groups (among each goose breed within groups), and within populations (individuals) (Table 1). Significant evidence from the six genes indicated that 50.2–90.86% of variances came from individuals, and only 9.14–49.8% of variances came from groups and populations. Compared with the other genes, no significant variance from the groups was observed for *EVC2P*, whereas the variance percentages from groups and populations were nearly equal for *COX2* and *COX3*. On the contrary, the variance from groups was larger than that from populations for *HBA1*, *HBB*, and especially *HBA2*, which also suggested apparent variance between the Chinese geese group and the Landaise geese.

**Table 1:** Results of the hierarchical AMOVA for grouping hypothesis^a^.

Gene	Source of variation	df	Sum of squares	Variance components	Fixation indices	Percentage of variation
*COX2*	Among groups	1	23.034	0.22963 Va	FCT:0.07741*	7.74
	Among populations within groups	6	43.931	0.23424 Vb	FSC:0.08558***	7.9
	Within populations	164	410.436	2.50266 Vc	FST:0.15637***	84.36
*COX3*	Among groups	1	3.828	0.03537 Va	FCT:0.04616*	4.62
	Among populations within groups	6	8.457	0.03467 Vb	FSC:0.04744*	4.52
	Within populations	164	114.169	0.69615 Vc	FST:0.09141***	90.86
*HBA1*	Among groups	1	83.622	0.92493 Va	FCT:0.16641*	16.64
	Among populations within groups	6	122.829	0.80916 Vb	FSC:0.17464***	14.56
	Within populations	164	627.142	3.82404 Vc	FST:0.31199***	68.8
*HBA2*	Among groups	1	101.254	1.32442 Va	FCT:0.36502*	36.5
	Among populations within groups	6	69.116	0.47080 Vb	FSC:0.20435***	12.98
	Within populations	164	300.636	1.83315 Vc	FST:0.49477***	50.52
*HBB*	Among groups	1	135.043	1.56541 Va	FCT:0.21460*	21.46
	Among populations within groups	6	170.257	1.15701 Vb	FSC:0.20195***	15.86
	Within populations	164	749.851	4.57226 Vc	FST:0.37321***	62.68
*EVC2P*	Among groups	1	32.043	0.01941 Va	FCT:0.00311	0.31
	Among populations within groups	6	177.216	1.19113 Vb	FSC:0.19147***	19.09
	Within populations	164	824.898	5.02987 Vc	FST:0.19398***	80.6

^a^ *Anser anser* and Landes geese comprise one group, and *Anser cygnoides* and Chinese domestic geese comprise the other group.

Va, variation among groups; Vb, variation among populations within groups; Vc, variation among populations.

* Significant (*P* < 0.05) and

***extremely significant values (*P* < 0.001).

**Fig 4:**
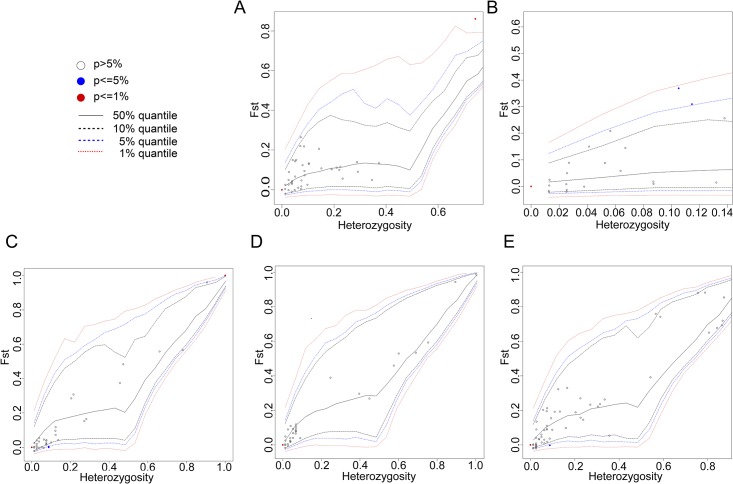
Differentiated loci between partial Chinese domestic geese and Landes geese. (A) *COX2*, (B) *COX3*, (C) *HBA1*, (D) *HBA2*, and (E) *HBB*. *F*_*ST*_: locus-specific genetic divergence among populations. Heterozygosity: measure of heterozygosity per locus. Single-locus *F*_*ST*_ values are plotted against heterozygosity, with the red-filled circles representing loci significant at the 1% level, and blue-filled circles representing loci significant at the 5% level.

We then screened the outliers at the SNP level to further evaluate the variance between the Chinese geese group and the Landaise geese. An extremely significant outlier locus (268) was found in the *COX2* gene, and two extremely significant outlier loci (3 and 696) were found in the *COX3* gene (Fig 4a and 4b). We also identified an extremely significant outlier locus (179) and two significant outlier loci (2 and 7) in the *HBA1* gene (Fig 4c). A significant outlier locus (107) in the *HBB* gene was also detected (Fig 4e). These results also illustrated the variance between the two geographical groups.

### Test for selection

The possible influence of artificial selection on the above-mentioned genes was first examined by Tajima’s *D* test and Fu and Li’s *D* test (S4 Table). Nearly all the significant values were found to be negative in our analysis. Test values examined by the two methods were all significantly negative for the *COX2*, *COX3*, and *HBA1* genes of descendants of *A*. *cygnoides* (DAC; including the S, SC, Z, and Zi populations). For the *HBA1* gene, both Tajima’s *D* and Fu and Li’s *D* tests were found to be significant for S, Zi, H, DAC, and descendants of *A*. *anser* (DAA: including the G and H populations). For the *HBA2* gene, only the G population was found to be significantly negative by both methods. Only Fu and Li’s *D* test detected significant negativity for the *HBB* gene in DAC. Except for the B and G populations, we detected significantly negative values for Fu’s *Fs H* test in all the other populations (S4 Table). To distinguish the effects of population dynamics and direct selection, we next employed the HKA neutrality test to compare the divergence of the candidate genes from the neutral *EVC2P* pseudogene. The HKA test results showed that only the *COX2* gene in the SC population (3.906, *p <* 0.05) was significantly different (Table 2).

**Table 2:** Values of the Hudson-Kreitman-Aguade test for each gene across populations^a^.

Population	*COX2*	*COX3*	*HBA1*	*HBA2*	*HBB*
B	0.024	N	0.007	0.049	N
DAC	1.537	1.247	0.153	0.323	1.917
S	0.617	0.958	0	0.478	2.306
SC	**3.906**	1.840	0.035	0.303	2.446
Z	1.932	1.761	0.011	0.16	0.919
Zi	0.055	N	1.461	0.414	1.454
Hui	0.000	0.003	0.007	0.02	0.049
DAA	0.379	0.006	0.96	0.048	0.189
G	0.843	0.527	0.695	0.153	0.068
H	0.144	0.018	1.271	0.035	0.297

^a^ HKA values with a significant *P*-value (*P*<0.05) in bold font; N indicates that data could not be calculated.

Historically, relaxation of functional constraints has been considered to be the main player during animal domestication. To evaluate whether relaxed selection had acted on these genes, we calculated the *d*_*N*_/*d*_*S*_ value of the protein coding region for each gene (S5 Table). Significant purifying selection was discovered in the DAC population at *COX2*, and in the DAC, Z, DAA, and H populations at *COX3*. With regard to the three nuclear genes, a significant accumulation of nonsynonymous mutations was found in *HBA1* in the Hui population and in *HBB* in the Zi population, which indicated relaxation of selective pressure. Interestingly, no synonymous mutation was found in the *HBB* gene in any population. In summary, the two mitochondrial genes seemed to undergo purifying selection, whereas the three nuclear genes showed signs of relaxed selection.

## Discussion

### Population variance between European domestic geese and Chinese domestic geese

It is widely believed that Chinese domestic geese originated from *A*. *cygnoides* and European domestic geese originated from *A*. *anser*. However, most of the evidence supporting this belief originated from maternal phylogenetic studies involving sequencing of a control region of mtDNA. This means that those results lack evidence at the nuclear and population genetics levels. In this study, we analyzed two mitochondrial genes and three nuclear genes from two wild ancestors (*A*. *cygnoides* and *A*. *anser*), Landaise geese (G and H populations), and four breeds of Chinese domestic geese (SC, S, Z, and Zi populations). Both the haplotype networks and the pairwise *F*_*ST*_ values of *HBA1*, *HBA2*, and *HBA3* showed a clear divergence between the European geese and the Chinese geese, indicating an independent genetic structure for the two goose populations (Figs 2 and 3). In addition, for the *HBA1*, *HBA2*, and *HBA3* genes, AMOVA supported our finding of larger variations between groups than between populations within groups. This means that apart from the variation between individuals, the divergence between the European geese and the Chinese geese was larger than that found within the varieties of geese. That is, our results suggest a distinct genetic variance between European geese and Chinese geese, which is consistent with the results of Shi et al. [21]. Compared with the three nuclear genes, the two mitochondrial genes appeared to be more conserved, which could be attributed to the more elastic spatial structure of the Hb protein as compared with the structure of COX. The haplotype network of *COX3* was significantly different between the two main geese groups, which may be due to its functional constraint or to gene introgression between the two ancestral groups as we previously reported [29]. The present study found molecular evidence of population variance between the Landaise geese and partial Chinese domestic geese, which supported the theory of an independent origin for European and Chinese domestic geese. However, the limited number of samples and genes evaluated in our study prevented a more comprehensive understanding of the origin of these groups of geese, and thus more targeted studies are needed.

### Signatures of positive selection and relaxed selection

The flyability of wild geese is the primary barrier preventing their early domestication owing to their limited feeding options, which makes the domestication of geese difficult. Unexpectedly, the domestication of wild geese was found to have occurred later than that of most other domestic animals, which suggested to us that intense artificial selection may have resulted in the degeneration of flyability [3]. As most studies have reported, the availability of energy directly affects the ability of birds to fly and always generates some molecular evidence [16, 30]. In this study, we worked under the assumption that energy metabolism and O_2_ transportation played an important role in the loss of flyability among domestic geese. Hence, we focused our study on Hb and the terminal oxidase of the mitochondrial respiratory chain. In our analysis, the molecular diversity of the five functional genes studied was much less than that of *EVC2P*; in fact, *EVC2P* had an appearance characteristic of a pseudogene that nearly evolved neutrally (Fig 1, Table 1, and S3 Table). These results are consistent with those of a previous report [31] evaluating pigs, which claimed that a population bottleneck could not explain the limited diversity of five candidate genes. It was suggested that those five genes suffered from selection pressure. Thus, we performed the neutral test for further verification, and evidence of both purifying selection and population expansion was discovered (S4 Table). With the increase in the human population, the number of domestic animals, including geese, has multiplied rapidly during recent years [5]. The results of the HKA test were obviously different from those of the neutral test, in that a statistically significant difference was found only in the *COX2* gene of the SC population. Under the stress of artificial selection, some neutral polymorphisms in the ancestral population may suddenly become advantageous; therefore, the beneficial alleles had an initial frequency (designated *p*) in the progenitor of the domesticated species. According to the report by Innan and Kim [32], *p* has a strong effect on the likelihood of detecting the signature of selection during domestication. When *p* was relative large, the HKA test became robust at detecting selection, which might explain the bias between the HKA and neutral tests.

Finally, we calculated the *d*_*N*_/*d*_*S*_ values to better understand the selection involved and were able to detect the signature of purifying selection in the *COX2* and *COX3* genes. In the study of Shen et al. [16], *COX3* was found to vary significantly between strongly and weakly locomotive birds [16]. However, with the degeneration of flyability, *COX3* showed relaxed selection in that study, which is in contrast to our results. It should be noted that the samples used in the two studies were quite different from one another. Shen et al. [16] evaluated various species of birds, most of which were under natural selection, whereas we concentrated on domestic geese that were under the influence of artificial selection. In a recent report, Wang et al. [33] found that positive selection, rather than relaxation of functional constraints, drove the degeneration of vision during chicken domestication. Historically, both vision and flyability were crucial for a bird’s survival. Therefore, the results of that study suggested to us that positive artificial selection played an important role in the degeneration of flyability in domestic geese. Interestingly, although most of the *P*-values in this study were not significant, a clear trend of relaxed selection was found in the *HBB* gene, which is consistent with the results of Shen et al. [16]. That is, with the degeneration of flyability, some functional genes accumulated more nonsynonymous than synonymous nucleotide substitutions, which indicates a relaxation of selective constraints.

Flight is one of the most crucial abilities affecting a bird’s viability, including its ability to mate, forage, and avoid predators. Unlike their wild ancestors, domestic geese living under a predictable farm environment have more accessibility to food and safety. These interventions by humans have reduced the pressure for flight in domestic geese, which may have eventually led to the degeneration of flyability. Unlike other traits, flight is a characteristic trait affecting survival and is hard to lose under natural selection. From the results of this study, we speculate that both positive artificial selection and relaxed selection have affected the ability of domestic geese to fly. Presumably, the progenitors of domestic geese that possessed weaker flyability were more easily caught, reared, and domesticated. As suggested by Shen et al. [34], genes encoding oxidative phosphorylation may have been sensitive to flight and suffered from positive artificial selection, whereas other genes related to energy metabolism, such as Hb-coding genes, may have been affected by the relaxation of functional constraints at a later time.

Loss of flight is considered a classic case of Darwin’s natural selection theory. So far, 26 families of birds in 17 different orders have been found to have lost the ability to fly, with obvious limb modifications. Recently, Burga et al. [35] made a breakthrough in analyzing the flightlessness mechanism of birds. They detected significant positive selection and found that the combined effects of variants in genes related to cilia or Hedgehog (Hh) signaling may be responsible for the reduction in growth of both the keel and wings in *Phalacrocorax harrisi*, leading to the bird’s flightlessness. They concluded that positive selection had played an important role in the fixation of the variants associated with loss of flight in birds with apparent limb modifications. However, these studies about flightless birds have seriously ignored another situation; namely, flightlessness without limb modifications, of which domesticated geese are a distinct example. Therefore, the findings in our study provide good supplemental information to present knowledge about the molecular mechanisms of flightlessness in birds. Unfortunately, even though we showed evidence of artificial selection, the limited number of genes analyzed and samples obtained did not provide comprehensive information about the mechanism behind the degeneration of flyability in domestic geese. Targeted studies will be helpful to supplement our hypothesis.

## Conclusions

Our study provides preliminary insight into the selection pressure acting on the degeneration of flyability in domestic geese. Combined with the results of previous studies, our findings support the independent origin of partial European domestic geese and Chinese domestic geese. It appears that both positive artificial selection and the relaxation of functional constraints played an important role in the degeneration of flyability in domestic geese.

## Supporting information

S1 Table Summary of sample collection for this study. (XLSX)

S2 Table Primers used to amplify the *COX2* and *COX3* sequences, and to provide partial sequences of *HBA1*, *HBA2*, and *HBB*. (XLSX)

S3 Table Nucleotide diversity of each gene across populations. (XLSX)

S4 Table Results of the neutral test based on Tajima’s *D*, Fu and Li’s *D*, and Fu’s *Fs H* tests. Statistically significant values are marked with *, **, or ***, and N indicates data that could not be calculated.
(XLSX)

S5 Table Estimates of the evolutionary divergence and positive selection for each gene. Analyses of the numbers of synonymous (*d*_*S*_) and nonsynonymous (*d*_*N*_) substitutions per site were conducted using the Nei-Gojobori method in MEGA 6. Standard error estimates were obtained using a bootstrap procedure (1,000 replicates). All positions containing alignment gaps and missing data were eliminated. *P*-values were calculated using the codon-based *Z* test with the alternative hypothesis of *d*_*N*_ > *d*_*S*_. Significant *P*-values are in bold font; N indicates data that could not be calculated.
(XLSX)

S1 File Amino acid mutations of *COX2*. (PDF)

S2 File Amino acid mutations of *COX3*. (PDF)

S3 File Amino acid mutations of *HBA1*. (PDF)

S4 File Amino acid mutations of *HBA2*. (PDF)

S5 File Amino acid mutations of *HBB*. (PDF)

## Abbreviations

AMOVA: analysis of molecular variance
B: *Anser cygnoides*
bce: Before the Common Era
COX: Cytochrome *c* oxidase
EVC2P: class II-related endogenous retrovirus
G: gray plumage Landes goose
H: spotted feather Landes goose
Hb: hemoglobin
*HBA1*: gene coding for α^D^
*HBA2*: gene coding for α^A^
*HBB*: gene coding for β^A^
HKA: Hudson-Kreitman-Aguade test
Hui: *Anser anser*
S: Lion-head goose
SC: Sichuan white goose
Z: Eastern Zhejiang white goose
Zi: Zi goose
