# Supplementary material for: Contribution of both positive selection and relaxation of selective constraints to degeneration of flyability during geese domestication
Source: PLoS One. 2017 Sep 25;12(9):e0185328. doi: 10.1371/journal.pone.0185328 (PMC5612694; doi:10.1371/journal.pone.0185328)
Supplement: S1 File — (PDF) [file pone.0185328.s006.pdf]

```
#MEGA
!COX2;
!Format
   DataType=Protein
   NSeqs=171 NSites=22
   Identical=. Missing=? Indel=-;


!Domain=Data;
[                               11222 22]
[                   1122 2237846012 22]
[             2356792515 7830839694 58]
#NC_011196    ANSQLFAPVD ALAIKIVMEL LS
#B1           .......... ....E..... ..
#B2           .......... .......... ..
#B3           .......... .......... ..
#B4           .......... ....E..... ..
#B5           .......... ....E..... ..
#B6           .......... .......... ..
#B7           .......... .......... ..
#B8           .......... ....E..... ..
#B9           .......... .......... ..
#Hui1         .......... ....E..... ..
#Hui2         .......... ....E..... ..
#Hui3         .......... ....E..... ..
#Hui4         .......... ........K. R.
#Hui5         .....L.... ....E..... ..
#Hui6         ....V..... .......... ..
#Hui7         .......... ....E..... ..
#G1           .......... .......... ..
#G2           .......... .......... ..
#G3           .......... .......... ..
#G4           .......... .......... ..
#G5           .......... .......... ..
#G6           .......... .......... ..
#G7           .......... .......... ..
#G8           .......... .......... ..
#G9           .......... .......... ..
#G10          .......... .......... ..
#G11          ....V..... .......... ..
#G12          .......... .......... ..
#G13          .......... .......... ..
#G14          ....V..... .......... ..
#G15          .......... .......... ..
#H1           S.W.V..... .......... ..
#H2           .......... ....E..... ..
#H3           .......... ....E..... ..
#H4           VTWE...S.. GV..E..... ..
#H5           F..EV..S.A .......... ..
#H6           .......... .V........ ..
#H7           VTWE...SI. ....EV...P R.
#H8           .......... .......... ..
#H9           .......S.. ....E..... R.
#H10          P......... .V........ ..
#H11          VTWE....I. ....EV.... ..
#H12          .......... .......... ..
#H13          .......... .......... ..
#H14          .......S.. .......... RL
#H15          .......... .......... ..
#H16          ........I. ....EV.... ..
#H17          .......... .......... ..
#H18          .......... .......... ..
```

```
#H19           .......... .......... ..
#H20           .......... .......... ..
#H21           .......... .......... ..
#H22           .......... .......... ..
#H23           .......... .......... ..
#H24           V......... .V........ ..
#S1            .......... ....E..... ..
#S2            .......... ....E..... ..
#S3            .......... ....E..... ..
#S4            .......... ....E..... ..
#S5            .......... ....E..... ..
#S6            .......... ....E..... ..
#S7            .......... ....E..... ..
#S8            .......... ....E..... ..
#S9            .......... ....E..... ..
#S10           .......... ....E..... ..
#S11           .......... ....E..... ..
#S12           .......... ....E..... ..
#S13           .......... ....E..... ..
#S14           .......... ....E..... ..
#S15           .......... ....E..... ..
#S16           .......... ....E..... ..
#S17           .......... ....E..... ..
#S18           .......... ....E..... ..
#S19           .......... ....E..... ..
#S20           .......... ....E..... ..
#S21           .......... ....E..... ..
#S22           ....VL.... ....E..... ..
#S23           .......... ....E..... ..
#S24           .......... ....E..... ..
#S25           .......... ....E..... ..
#S26           .......... ....E..... ..
#S27           .......... ....E..... ..
#S28           .......... ....E..... ..
#S29           .......... ....E..... ..
#S30           .......... ....E..... ..
#S31           .......... ....E..... ..
#S32           .......... ....E..V.. ..
#S33           .......... ....E..... ..
#S34           .......... ....E..... ..
#S35           .......... ....E..... ..
#S36           .......... ....E..... ..
#S37           .......... ....E..... ..
#S38           .......... ....E..... ..
#S39           .......S.. ....E..... ..
#S40           .......... .......... ..
#SC1           .......... ....E..... ..
#SC2           .......... ....E..... ..
#SC3           STW.VL.S.. .M..E..... ..
#SC4           VTW.VLPF.. ....E..... ..
#SC5           ..W....S.. ....E..... ..
#SC6           ....V..... ....E..... ..
#SC7           .......... ....E..... ..
#SC8           .......S.. ....E..... ..
#SC10          .......... ....E..... ..
#SC11          .......... ....E..... ..
#SC12          ..W.V..... ....E..... ..
#SC13          .......... ....E..... ..
#SC14          .......S.. ....E..... ..
#SC15          ..W..LPS.. VV..E..... ..
#SC16          .......... ....E..... ..
#SC18          .......... ....E..... ..
```

```
#SC19          .......... ....E..... ..
#SC20          .......... ....E..... ..
#Z1            V.W.VL.S.. .V..E..... ..
#Z2            ..W....... ....E..... ..
#Z3            ....V..... ....E..... ..
#Z4            .......... ..TVEVI... ..
#Z5            .......... ....E..... ..
#Z6            .......... ....E..... ..
#Z7            S.W....S.. ....E..... ..
#Z8            ..W.V..S.. ....E..... ..
#Z9            S.W..L.Y.. .V..E..... ..
#Z10           .......... ..TVEVI... ..
#Z11           .......... ....E..... ..
#Z12           .......... ....E..... ..
#Z13           .......... ....E..... ..
#Z14           ..W.V..... ....E..... ..
#Z15           .......... ....E..... ..
#Z16           .......... ....E..... ..
#Z17           .......... ....E..... ..
#Z18           .......... ....E..... ..
#Z19           .......... ....E..... ..
#Z20           .......... ....E..... ..
#Z21           .......... ....E..... ..
#Z22           .......S.. ....E..... ..
#Z23           .......... ....E..... ..
#Z24           .......... ....E..... ..
#Z25           .......... ....E..... ..
#Z26           .......... ....E..... ..
#Z27           .......... ....E..... ..
#Z28           .......... ....E..... ..
#Z29           .......... ....E..... ..
#Z30           .......... ..TVEVI... ..
#Z31           .......... ....E..... ..
#Z32           .......... ....E..... ..
#Z33           .......... ....E..... ..
#Z34           .......... ....E..... ..
#Z35           .......... ....E..... ..
#Zi1           .......... ....E..... ..
#Zi2           .......... ....E..... ..
#Zi3           .......... ....E..... ..
#Zi4           .......... ....E..... ..
#Zi5           .......... ....E..... ..
#Zi6           .......... .......... ..
#Zi7           .......... ....E..... ..
#Zi8           .......... ....E..... ..
#Zi9           .......... ....E..... ..
#Zi10          .......... ....E..... ..
#Zi11          .......... ....E..... ..
#Zi12          .......... .......... ..
#Zi13          .......... ....E..... ..
#Zi14          .......... ....E..... ..
#Zi15          .......... ....E..... ..
#Zi16          .......... ....E..... ..
#Zi17          .......... ....E..... ..
#Zi18          .......... ....E..... ..
#Zi19          .......... ....E..... ..
#Zi20          .......... ....E..... ..
#Zi21          .......... ....E..... ..
#Zi22          .......... ....E..... ..
```
