# Supplementary material for: Contribution of both positive selection and relaxation of selective constraints to degeneration of flyability during geese domestication
Source: PLoS One. 2017 Sep 25;12(9):e0185328. doi: 10.1371/journal.pone.0185328 (PMC5612694; doi:10.1371/journal.pone.0185328)
Supplement: S2 File — (PDF) [file pone.0185328.s007.pdf]

```
#MEGA
!COX3;
!Format
   DataType=Protein
   NSeqs=173 NSites=7
   Identical=. Missing=? Indel=-;


!Domain=Data;
[                  12]
[                 456]
[             1245110]
#NC_011196    MAQAMIG
#B1           .......
#B2           .......
#B3           .......
#B4           .......
#B5           .......
#B6           .......
#B7           .......
#B8           .......
#B9           .......
#Hui1         .......
#Hui2         .......
#Hui3         .......
#Hui4         ......R
#Hui5         .......
#Hui6         .......
#Hui7         .......
#G1           .......
#G2           .......
#G3           .......
#G4           .......
#G5           .......
#G6           .......
#G7           .......
#G8           .......
#G9           .......
#G10          .......
#G11          .......
#G12          .......
#G13          .......
#G14          .......
#G15          .......
#H1           .......
#H2           .......
#H3           .......
#H4           .......
#H5           .......
#H6           .......
#H7           ....V..
#H8           .......
#H9           .......
#H10          .......
#H11          ....V..
#H12          .......
#H13          .......
#H14          .......
#H15          .......
#H16          ....V..
#H17          .......
#H18          .......
```

#H19	....
#H20	....
#H21	....
#H22	....
#H23	....
#H24	....
#S1	....
#S2	....
#S3	....
#S4	....
#S5	....
#S6	....
#S7	....
#S8	....
#S9	....
#S10	....
#S11	....
#S12	.....M.
#S13	....
#S14	....
#S15	....
#S16	....
#S17	....
#S18	....
#S19	....
#S20	....
#S21	....
#S22	....
#S23	V.H....
#S24	..H....
#S25	L......
#S26	L......
#S27	..HT...
#S28	..HT...
#S29	..H....
#S30	....
#S31	V......
#S32	....
#S33	....
#S34	....
#S35	....
#S36	....
#S37	....
#S38	....
#S39	....
#S40	....
#SC1	....
#SC2	V.PT...
#SC3	....
#SC4	....
#SC5	....
#SC6	....
#SC7	....
#SC8	....
#SC9	....
#SC10	....
#SC11	....
#SC12	....
#SC13	....
#SC14	....
#SC15	....
#SC16	....

#SC17	....
#SC18	....
#SC19	....
#SC20	....
#Z1	..H....
#Z2	....
#Z3	....
#Z4	V.H....
#Z5	..H....
#Z6	..H....
#Z7	....
#Z8	V......
#Z9	....
#Z10	....
#Z11	....
#Z12	....
#Z13	....
#Z14	....
#Z15	....
#Z16	....
#Z17	....
#Z18	....
#Z19	....
#Z20	....
#Z21	.P.....
#Z22	....
#Z23	....
#Z24	....
#Z25	....
#Z26	....
#Z27	....
#Z28	....
#Z29	....
#Z30	....
#Z31	....
#Z32	....
#Z33	....
#Z34	....
#Z35	....
#Zi1	....
#Zi2	....
#Zi3	....
#Zi4	....
#Zi5	....
#Zi6	....
#Zi7	....
#Zi8	....
#Zi9	....
#Zi10	....
#Zi11	....
#Zi12	....
#Zi13	....
#Zi14	....
#Zi15	....
#Zi16	....
#Zi17	....
#Zi18	....
#Zi19	....
#Zi20	....
#Zi21	....
#Zi22	....
