# Supplementary material for: Contribution of both positive selection and relaxation of selective constraints to degeneration of flyability during geese domestication
Source: PLoS One. 2017 Sep 25;12(9):e0185328. doi: 10.1371/journal.pone.0185328 (PMC5612694; doi:10.1371/journal.pone.0185328)
Supplement: S3 File — (PDF) [file pone.0185328.s008.pdf]

```
#MEGA
!HBA1;
!Format
   DataType=Protein
   NSeqs=168 NSites=12
   Identical=. Missing=? Indel=-;


!Domain=Data;
[                11 11]
[             25533 33]
[        1234532813 47]
#B1      MLTADESHAA AA
#B2      .......... ..
#B3      .......... ..
#B4      .......... ..
#B5      .......... ..
#B6      .......... ..
#B7      L.I.F..... ..
#B8      .......... ..
#B9      .......... ..
#Hui1    .......... ..
#Hui2    ..N....... ..
#Hui3    .......... ..
#Hui4    L...Y..... ..
#Hui5    ..N....... ..
#Hui6    .......... ..
#Hui7    .......... ..
#G1      .......... ..
#G2      .......... ..
#G3      .......... ..
#G4      .......... ..
#G5      .......... ..
#G6      .......... ..
#G7      .......... ..
#G8      .......... ..
#G9      .......... ..
#G10     .......... ..
#G11     .......... ..
#G12     .......... ..
#G13     .......... ..
#G14     .......... ..
#G15     L......... ..
#H1      .......... ..
#H2      .P........ ..
#H3      .......... ..
#H4      ........P. ..
#H5      .......... ..
#H6      .......... ..
#H7      .......... ..
#H8      .......... ..
#H9      .......... ..
#H10     .......... ..
#H11     .......... ..
#H12     .......... ..
#H13     .......... ..
#H14     .......... ..
#H15     .......... ..
#H16     .......... ..
#H17     .......... ..
#H18     .......... ..
#H19     .......... ..
```

```
#H20      .......... ..
#H21      .......... ..
#H22      .......... ..
#H23      .......... ..
#H24      .......... ..
#S1       .......... ..
#S2       .......... ..
#S3       .........P ..
#S4       .......... ..
#S5       .......... ..
#S6       .......... ..
#S7       ..PV.K.... ..
#S8       .......... ..
#S9       .......... ..
#S10      .......... ..
#S11      .......... ..
#S12      .......... ..
#S13      ......F... ..
#S14      .......... ..
#S15      .......... ..
#S16      .......... ..
#S17      ......FP.. ..
#S18      .......... ..
#S19      .......... ..
#S20      .......... ..
#S21      .......... ..
#S22      ......F... ..
#S23      .......... ..
#S24      .......... ..
#S25      .......... ..
#S26      .......... ..
#S27      .......... ..
#S28      ......FP.. ..
#S29      .......... ..
#S30      .......... ..
#S31      .......... ..
#S32      .......... ..
#S33      .......... ..
#S34      .......... ..
#S35      .......... ..
#S36      .......... ..
#S37      .......... ..
#S38      .......... ..
#S39      .......... ..
#S40      .......... ..
#SC1      .......... ..
#SC2      .......... ..
#SC3      ..N....... ..
#SC4      .......... ..
#SC5      .......... ..
#SC6      .......... ..
#SC7      .......... ..
#SC8      .......... ..
#SC9      .......... ..
#SC10     .......... ..
#SC11     .......... ..
#SC12     .......... ..
#SC13     .......... ..
#SC14     .......... ..
#SC15     .......... ..
#SC16     .......... ..
#SC17     LAQ....... ..
```

```
#SC18    ..N....... ..
#SC20    .......... ..
#Z1      ..N....... ..
#Z2      .......... ..
#Z3      .......... ..
#Z4      ..N....... ..
#Z5      .......... ..
#Z6      .......... ..
#Z7      .......... ..
#Z8      .......... ..
#Z9      .......... ..
#Z10     .......... ..
#Z11     ..H....... ..
#Z12     K.N....... ..
#Z13     .......... ..
#Z14     .......... ..
#Z15     .......... ..
#Z16     ..N....... ..
#Z17     LSH....... ..
#Z18     .......... ..
#Z19     .......... ..
#Z20     .......... ..
#Z21     .......... ..
#Z22     .......... ..
#Z23     .......... ..
#Z24     LSH....... ..
#Z25     .......... ..
#Z26     .......... ..
#Z27     .......... ..
#Z28     .......... ..
#Z29     .......... ..
#Z30     .......... ..
#Z31     .......... ..
#Z32     LVN....... ..
#Z33     .......... ..
#Z34     .......... ..
#Z35     .......... ..
#Zi2     .......... ..
#Zi3     .......... ..
#Zi4     .......... ..
#Zi5     .......... ..
#Zi6     .......... ..
#Zi8     .......... ..
#Zi10    .......... ..
#Zi11    .......... ..
#Zi12    ......F... ..
#Zi13    .......... ..
#Zi14    .......... ..
#Zi15    .......... ..
#Zi16    .......... VV
#Zi17    .......... ..
#Zi18    .......... ..
#Zi19    .......... ..
#Zi20    .......... ..
#Zi21    .......... ..
#Zi22    .......... ..
```
