# Supplementary material for: Contribution of both positive selection and relaxation of selective constraints to degeneration of flyability during geese domestication
Source: PLoS One. 2017 Sep 25;12(9):e0185328. doi: 10.1371/journal.pone.0185328 (PMC5612694; doi:10.1371/journal.pone.0185328)
Supplement: S4 File — (PDF) [file pone.0185328.s009.pdf]

```
#MEGA
!HBA2;
!Format
   DataType=Protein
   NSeqs=167 NSites=3
   Identical=. Missing=? Indel=-;


!Domain=Data;
[         11]
[         01]
[        235]
#B1      VGP
#B2      ...
#B3      ...
#B4      ...
#B5      ...
#B6      ...
#B7      ...
#B8      ...
#B9      ...
#Hui1    ...
#Hui2    ...
#Hui3    ...
#Hui4    ...
#Hui5    ...
#Hui6    M..
#Hui7    ...
#G1      .V.
#G2      ...
#G3      ...
#G4      ...
#G5      ...
#G6      ...
#G7      ...
#G8      ...
#G9      ...
#G10     ...
#G11     ...
#G13     ...
#G14     ...
#G15     ...
#H1      ...
#H2      ...
#H3      ...
#H4      ...
#H8      ...
#H9      ...
#H10     ...
#H11     ...
#H12     ...
#H13     ...
#H14     ...
#H15     ...
#H16     ...
#H17     ...
#H18     ...
#H19     ...
#H20     ...
#H21     ...
#H22     ...
#H23     ...
```

```
#H24     ...
#S1      ...
#S2      ...
#S3      ...
#S4      ...
#S5      ...
#S6      ...
#S7      ...
#S8      ...
#S9      ...
#S10     ...
#S11     ...
#S12     ...
#S13     ...
#S14     ...
#S15     ...
#S16     ...
#S17     ...
#S18     ...
#S19     ...
#S20     ...
#S21     ...
#S22     ...
#S23     ...
#S24     ...
#S25     ...
#S26     ...
#S27     ...
#S28     ...
#S29     ...
#S30     ...
#S31     ...
#S32     ...
#S33     ...
#S34     ...
#S35     ...
#S36     ...
#S37     ...
#S38     ...
#S39     ...
#S40     ...
#SC1     ...
#SC2     ...
#SC3     L..
#SC4     ...
#SC5     ...
#SC6     ...
#SC7     ...
#SC8     ...
#SC9     ...
#SC10    ...
#SC11    ...
#SC12    ...
#SC13    ...
#SC14    ...
#SC15    ...
#SC16    ...
#SC17    ...
#SC18    ...
#SC19    ...
#SC20    L..
#Z1      ...
```

#Z2	...
#Z3	...
#Z4	...
#Z5	...
#Z6	...
#Z7	...
#Z8	...
#Z9	...
#Z10	...
#Z11	...
#Z12	...
#Z13	...
#Z14	...
#Z15	...
#Z16	...
#Z17	...
#Z18	...
#Z19	...
#Z20	...
#Z21	...
#Z22	...
#Z23	...
#Z24	...
#Z25	...
#Z26	...
#Z27	...
#Z28	...
#Z29	...
#Z30	...
#Z31	...
#Z32	...
#Z33	...
#Z34	...
#Z35	...
#Zi1	...
#Zi2	...
#Zi3	...
#Zi4	...
#Zi5	...
#Zi6	...
#Zi7	..S
#Zi8	...
#Zi9	...
#Zi10	...
#Zi11	...
#Zi13	...
#Zi14	...
#Zi15	...
#Zi16	...
#Zi17	...
#Zi18	...
#Zi19	...
#Zi20	...
#Zi21	...
#Zi22	...
