# Supplementary material for: Contribution of both positive selection and relaxation of selective constraints to degeneration of flyability during geese domestication
Source: PLoS One. 2017 Sep 25;12(9):e0185328. doi: 10.1371/journal.pone.0185328 (PMC5612694; doi:10.1371/journal.pone.0185328)
Supplement: S5 File — (PDF) [file pone.0185328.s010.pdf]

```
#MEGA
!HBB;
!Format
   DataType=Protein
   NSeqs=172 NSites=4
   Identical=. Missing=? Indel=-;


!Domain=Data;
[         557]
[        1674]
#B1      LAQR
#B2      ....
#B3      ....
#B4      ....
#B5      ....
#B6      ....
#B7      ....
#B8      ....
#B9      ....
#Hui1    ....
#Hui2    ....
#Hui3    ....
#Hui4    ....
#Hui5    ....
#Hui6    ....
#Hui7    ....
#G1      ....
#G2      ....
#G3      ....
#G4      ....
#G5      ....
#G6      ....
#G7      ....
#G8      ....
#G9      ....
#G10     ....
#G11     ....
#G12     ....
#G13     ....
#G14     ....
#G15     ....
#H1      ....
#H2      ....
#H3      ....
#H4      ....
#H5      ....
#H6      ....
#H7      ....
#H8      ....
#H9      ....
#H10     ....
#H11     ....
#H12     ....
#H13     ....
#H14     ....
#H15     ....
#H16     ....
#H17     ....
#H18     ....
#H19     ....
#H20     ....
```

#H21	....
#H22	....
#H23	....
#H24	....
#S1	....
#S2	....
#S3	....
#S4	....
#S5	....
#S6	....
#S7	....
#S8	....
#S9	....
#S10	....
#S11	....
#S12	....
#S13	....
#S14	....
#S15	....
#S16	....
#S17	....
#S18	....
#S19	....
#S20	....
#S21	....
#S22	....
#S23	....
#S24	....
#S25	....
#S26	....
#S27	....
#S28	....
#S29	....
#S30	....
#S31	....
#S32	....
#S33	....
#S34	....
#S35	....
#S36	....
#S37	....
#S38	....
#S39	....
#S40	....
#SC1	....
#SC2	....
#SC3	....
#SC4	....
#SC5	....
#SC6	....
#SC7	....
#SC8	....
#SC9	....
#SC10	....
#SC11	....
#SC12	....
#SC13	....
#SC14	....
#SC15	....
#SC16	....
#SC17	....
#SC18	....

#SC19	....
#SC20	....
#Z1	....
#Z2	....
#Z3	....
#Z4	....
#Z5	....
#Z6	....
#Z7	....
#Z8	....
#Z9	....
#Z10	....
#Z11	....
#Z12	....
#Z13	....
#Z14	....
#Z15	....
#Z16	....
#Z17	....
#Z18	....
#Z19	....
#Z20	....
#Z21	....
#Z22	....
#Z23	....
#Z24	....
#Z25	....
#Z26	....
#Z27	....
#Z28	....
#Z29	....
#Z30	....
#Z31	....
#Z32	....
#Z33	....
#Z34	....
#Z35	....
#Zi1	....
#Zi2	....
#Zi3	....
#Zi4	VPHG
#Zi5	....
#Zi6	....
#Zi7	....
#Zi8	....
#Zi9	....
#Zi10	....
#Zi11	....
#Zi12	....
#Zi13	....
#Zi14	....
#Zi15	....
#Zi16	....
#Zi17	....
#Zi18	....
#Zi19	....
#Zi20	....
#Zi21	....
#Zi22	....
